# The efficacy and safety of high-dose tranexamic acid in adolescent idiopathic scoliosis: a meta-analysis

**DOI:** 10.1186/s13018-020-02158-8

**Published:** 2021-01-14

**Authors:** Indra K. Shrestha, Tian-Yi Ruan, Lan Lin, Miao Tan, Xue-Qing Na, Qi-Cai Qu, Jian-Chun Chen, Yong-Yu Si, Jian-Ping Tao

**Affiliations:** Department of Anesthesiology, The Second Affiliated Hospital of Kunming Medical University, No.374 of Dianmian Avenue, Wuhua District, Kunming, 650101 Yunnan China

**Keywords:** Scoliosis surgery, Posterior spinal fusion, Tranexamic acid, Blood loss, Thromboembolic event

## Abstract

**Background:**

This study aimed to evaluate the efficacy and safety of using high-dose intravenous tranexamic acid (TXA) to reduce blood loss in idiopathic scoliosis surgery.

**Methods:**

This study was a meta-analysis, which consisted of retrospective cohort studies (RCSs) and randomized control trials (RCTs) found by searching electronic databases, namely PubMed, Web of Science, The Cochrane Central Register of Controlled Trials (CENTRAL), and the Google Scholar Database, dating from 1960 to 2019. The points of interest included total blood loss, a need for transfusion and transfusion criteria, surgery time, and the evidence of intraoperative and postoperative complications, such as seizures or thromboembolic events. The weighted mean differences (WMD) and 95% confidence interval (CI) of blood loss in the TXA intervention group compared to the control or placebo group were extracted and combined using the random effects model.

**Results:**

In this meta-analysis, there was a total of three RCSs and two RCTs, which involved 334 patients. The results showed that blood loss is significantly reduced, with a weighted mean difference in the TXA group (WMD = − 525.14, *P* = 0.0000, CI ranged from − 839.83, − 210.44, *I*^2^ = 82%). Heterogeneity was assessed using the random effects model.

**Conclusions:**

A high dose of intravenous TXA reduced blood loss during adolescent idiopathic scoliosis surgery and did not lead to any significant thromboembolic event. Therefore, a high dose appears to be effective and safe for adolescent idiopathic scoliosis surgery. However, more high-quality research based on larger randomized controlled trials is still needed.

## Background

Posterior spinal fusion (PSF), an operation in which the spine is stabilized by grafting bone to fuse it, is a major procedure for the treatment of idiopathic scoliosis. A PSF involves a significant soft tissue dissection and bone cut, which can result in excessive bleeding and increases the time of the operation and the need for a blood transfusion. Often, for the above reasons, the surgeon and anesthetist are concerned about significant blood loss during the intraoperative and postoperative period. A blood transfusion may be allogeneic and autologous, but both have the side effects and reactions related to transfusion. The potential complications of allogeneic blood transfusions are as follows: infection (i.e., hepatitis B virus (HBV), hepatitis C virus (HCV), human immunodeficiency virus (HIV)), alloimmunization, transfusion-related acute lung injury, renal impairment or failure, transfusion-related circulatory overload, and transfusion-induced coagulopathy [1]. Thus, tranexamic acid (TXA) has been used to prevent the complications mentioned above and, also, to minimize the rate of morbidity and mortality associated with blood loss.

TXA is a synthetic antifibrinolytic that inhibits the activation of plasminogen to plasmin, thereby inhibiting binding to fibrin, which prevents fibrinolysis. It can reduce bleeding during scoliosis surgery and help to prevent inflammation and platelet degradation [2], according to individual studies as well as meta-analyses, which compared the efficacy and safety of low dose TXA with a placebo. TXA was first prescribed to women with heavy menstrual blood loss and patients with hereditary bleeding disorders [3]. It is used in pediatric, adult, and geriatric patients, and studies show it has been used in cardiac surgery, spine surgery, gynecological and obstetrics surgery, orthopedic surgery (knee and hip replacement), liver surgery, ENT surgery, and urological surgery [4–9]. Despite the several meta-analyses that have been done concerning the use of low dose TXA for posterior spinal fusion, there is a lack of studies of high dose TXA in adolescent idiopathic scoliosis surgery.

Recently, several researchers have used high doses of TXA to reduce intraoperative and postoperative bleeding during major operative procedures. Therefore, we conducted this meta-analysis to evaluate the effectiveness and safety of high-dose TXA in reducing the amount of blood loss and minimizing the transfusion of allogeneic blood in patients with adolescent idiopathic scoliosis surgery.

## Materials and methods

### Literature search strategy

In this meta-analysis, we searched PubMed, Web of Science, and The Cochrane Central Register of Control Trials to identify the relevant studies, including prospective and retrospective cohort studies (RCTs), review articles, meta-analyses, and randomized control trials (RCTs) carried out between 1960 and December 2019. The key words were as follows: scoliosis surgery, posterior spinal fusion, tranexamic acid, and high dose tranexamic acid. Different databases adopt different search strategies. Taking PubMed as an example, the search entry was (“Scoliosis Surgery [Mesh]” OR “Posterior spinal fusion”) AND (“tranexamic acid [Mesh]” OR “High dose tranexamic acid”). We used EndNote X4 (Duke University) to check for duplication and removed all duplication by using EndNote Medline (PC) and EndNote RIS.

### Criteria for inclusion in the study

The study design consisted of RCTs and RCSs. After a thorough evaluation, two RCTs and three RCSs were found that met the inclusion criteria. The basic characteristics of criteria for inclusion were as follows: (1) All patients should have undergone idiopathic scoliosis surgery; (2) the study should involve preoperative intravenous administration of a high dose ≥ 50 mg/kg bolus of tranexamic acid and an intraoperative maintenance dose of TXA, which was compared with a control group (i.e., one with no TXA or a placebo of normal saline); (3) all the studies should include reported outcomes of the following: total blood loss (perioperative), need for blood transfusion, either allogeneic or cell salvage, and complications during the intraoperative and postoperative period.

**Fig. 1:**
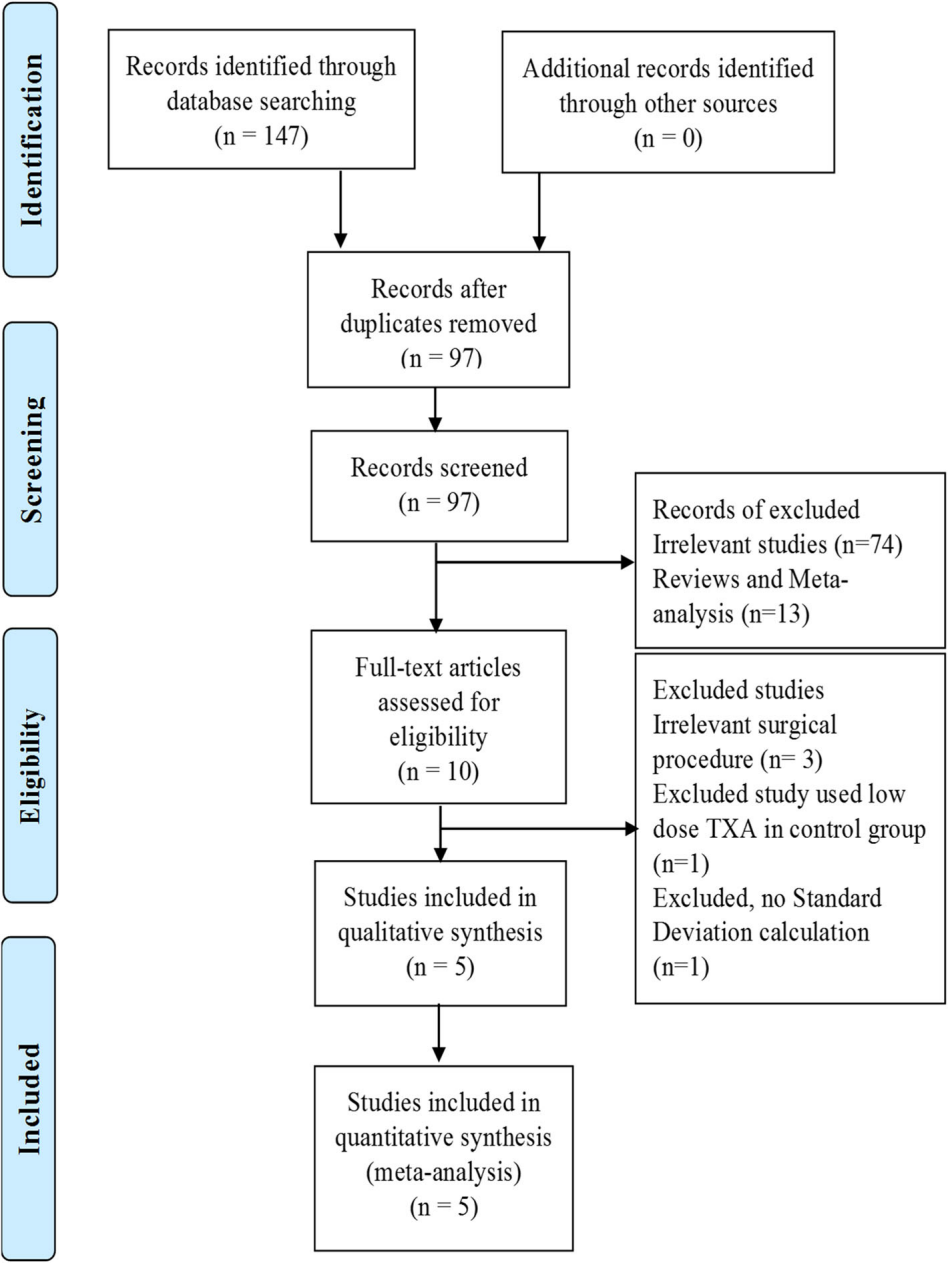
Method of selection for inclusion in the study

Other studies were excluded due to case reports, reviews (systematic and narrative), meta-analysis, no relevant intervention or procedures, comments, case-control design, and editorials. A flow chart of the study selection procedure is shown in Fig. 1, and the characteristics of the included studies are detailed in Table 1.

**Table 1:** Blood loss and Surgery time of included studies

Study	High dose tranexamic acid					Control/placebo				
	No. of patient	Blood loss (mL)	SD	Surgery time (h)	SD	No. of patient	Blood loss (mL)	SD	Surgery time (h)	SD
Ng et al. [10]	55	1826.11	1081.45	7.28	2.04	35	3889.6	2440.8	8.36	1.4
da Rocha et al. [11]	21	1097.3	323.9	4	1.2	19	1406.5	372.1	4.3	1.4
Sethna et al. [12]	23	1072	425	6.6	1.8	21	1420	644	6.1	1.8
Lykissas et al. [13]	25	537	320	NR	NR	24	1245	896	NR	NR
Goobie et al. [14]	56	836	373	4.5	1.2	55	1031	484	4.2	0.96

*NR* not reported, *h* hour, *SD* standard deviation

### Data extraction

The included data were extracted independently by two authors, and the features of each study were recorded with their outcomes. The general features of the included studies were as follows: author names, patient demographics, types of study design, publication date, type of surgical procedure and anesthesia method, intervention (i.e., intravenous TXA and control with no TXA or placebo with normal saline), need for transfusion (i.e., allogeneic blood, packed blood cell, cell salvage) during intraoperative and postoperative periods after measuring Hb levels, and the measurements of outcomes, with or without complications.

### The quality assessment

The quality assessment of the included RCTs followed the recommendations of the Cochrane Handbook for Systematic Reviews, consisting of random sequence generation, allocation concealment, blinding, incomplete outcome data, selective reporting, and other bias [15]. The quality assessment of the included RCSs comprised measuring exposure or non-exposed measuring outcomes, follow-up, analysis of the cohort, confounding bias, strengths (multiple, multiple exposures), and weaknesses (cost and time, prone to bias). The Newcastle-Ottawa Scale (NOS) was used to assess the quality of the nonrandomized studies from three broad perspectives, namely the selection of the study groups, the comparability of the groups, and ascertaining either exposure or outcome of interest. The included studies have an NOS score higher than 5, which signifies a good quality study.

### Subgroup analysis

Only five studies were included in this meta-analysis, and due to the limited sample size, the subgroup analysis was difficult to complete. This was because when we tried to conduct the subgroup analysis according to the intervention level, only one study used 50 mg/kg TXA, and the other four studies used 100 mg/kg TXA.

### The main points of interest

In this study, the main points of interest were total blood loss, surgery time, and the evidence of intraoperative and postoperative complications.

#### The total blood loss

The total blood loss was defined as the total blood loss during the surgery, which was evaluated during the surgery. It was measured by counting all sponges and tapes and weighing them accurately by standard measurement, measuring the amount of blood collected in cell salvage and intraoperative suction drainage and subtracting the fluids irrigated during surgery, including the irrigation in the surgical field. The clinical significance was the fact that this index can be used to evaluate the extent of surgical trauma.

#### The surgery time

The surgery time was defined as the duration of the whole surgery, which was evaluated from the beginning of the operation, being the given time of incision, to closure of skin, at the end of the operation. This index can be used to evaluate the degree of difficulty of the operation.

#### Complications

In this study, the intraoperative and postoperative complications were defined as the adverse reactions that occurred during and after surgery, such as seizure, thromboembolic events, infection, kidney failure, arterial occlusion, or gastrointestinal dysfunction. This index can be used to evaluate the complications of the operation.

### Statistical methods

We conducted the statistical analysis by using STATA 15.0 and Review Manager RevMan version 5.3. The means and standard deviations were pooled from all included studies to measure the weighted mean difference (WMD) of blood loss and operative time, comparing the tranexamic acid group with the control or placebo group. Dichotomous outcomes were analyzed using relative risk (RR) and 95% confidence intervals (CIs). A random effects model or fixed effects model was used to pool the data, and the statistical heterogeneity between summary data was assessed by the chi-squared test, and its extent was quantified by the *I*^2^ statistic. The fixed effects model was employed when there was no evidence of heterogeneity, *I*^2^ ≤ 50%. A *P* < 0.05 was considered to indicate statistical significance in this meta-analysis.

## Results

### Literature search results

Based on a literature search of electronic databases, initially, a total of 147 articles were identified as matching the inclusion criteria: PubMed (*n* = 66), Web of Science (*n* = 80), and the Cochrane Library (*n* = 1). After reviewing the articles, we excluded all irrelevant and duplicate articles, and finally focused on five studies [10–14] of RCSs and RCTs, most of which were published recently, and one of which was published in 2005 [12].

### General characteristics

**Fig. 2:**
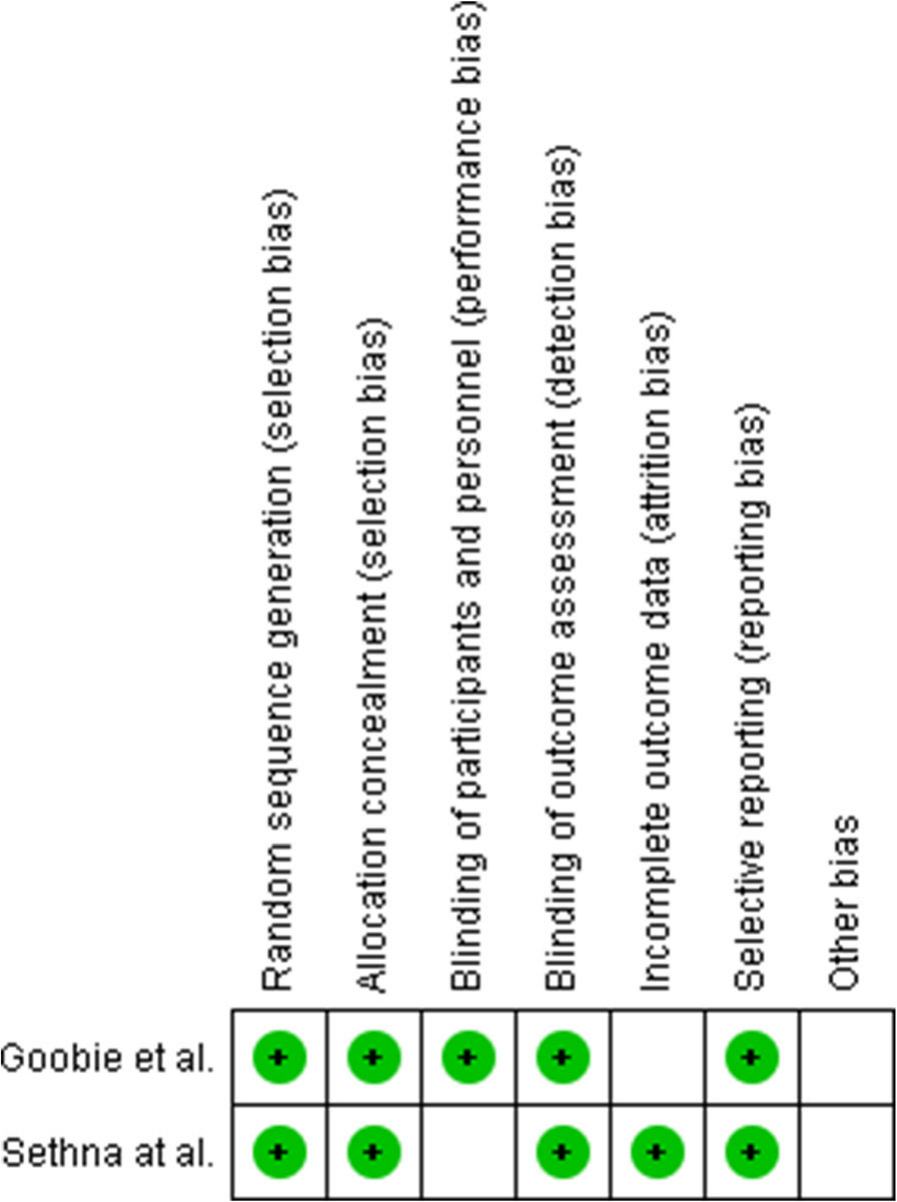
Bias risk assessments for each RCT study (“+” indicates present or yes, and “blank” indicates unclear)

A total of 334 patients participated in the studies for meta-analysis. There were 180 patients in the TXA group and 154 patients in the control group. In four studies [10–13], a dose of 100 mg/kg was used, bolus ranging from 15–30 min IV, and in one study [14], a dose of 50 mg/kg bolus was used. The participants were patients undergoing adolescent scoliosis surgery. The intervention in the TXA group was a high dose of TXA while the intervention in the control group was 0.9% normal saline or no TXA. The maintenance dose of TXA, from the beginning of surgery to skin closure, was 10 mg/kg/h in four of the studies [10–14], and 30 mg/kg/h in the other study [11] (Tables 1 and 2). The bias risk assessment for each RCT study is shown in Fig. 2.

**Table 2:** Characteristics of the included studies

Study	Age		No. of patients		Study design	Participants	Intervention	Comparison	Level of fused		Transfusion criteria	Surgery	Body weight (kg)	
	TXA	C	TXA	C			TXA	C	TXA	C			TXA	C
Ng et al. [10]	15.16	15.3	55	35	RCS	Patients received ASS	100 mg/kg + 10 mg/kg/h	No TXA	13.5	12.1	Hb < 8 g/dL	PSF	45.25	42.88
da Rocha et al. [11]	18	21.6	21	19	RCS	Patients received ASS	100 mg/kg + 30 mg/kg/h	No TXA	9.4	9.2	Not reported	PSF	55.5	51.8
Sethna et al. [12]	13.6	14	23	21	RCT	Patients received ASS	100 mg/kg + 10 mg/kg/h	NS 0.9%	14	13	Hb < 9 g/dL	PSF	59.4	52.4
Lykissas et al. [13]	14.7	13.5	25	24	RCS	Patients received ASS	100 mg/kg + 10 mg/kg/h	No TXA	10.7	12.6	Hb < 7 g/dL	PSF	Not reported	
Goobie et al. [14]	14.9	14.7	56	55	RCT	Patients received ASS	50 mg/kg + 10 mg/kg/h	NS 0.9%	10	9	Hb ≤7.3 g/dL	PSF	55.1	51.6

*TXA* tranexamic acid, *C* control, *PSF* posterior spinal fusion, *NS* normal saline, *RCS* retrospective cohort studies, *RCT* randomized control trials, *ASS* adolescent scoliosis surgery

### The effects of TXA in reducing blood loss in scoliosis surgery

**Fig. 3:**
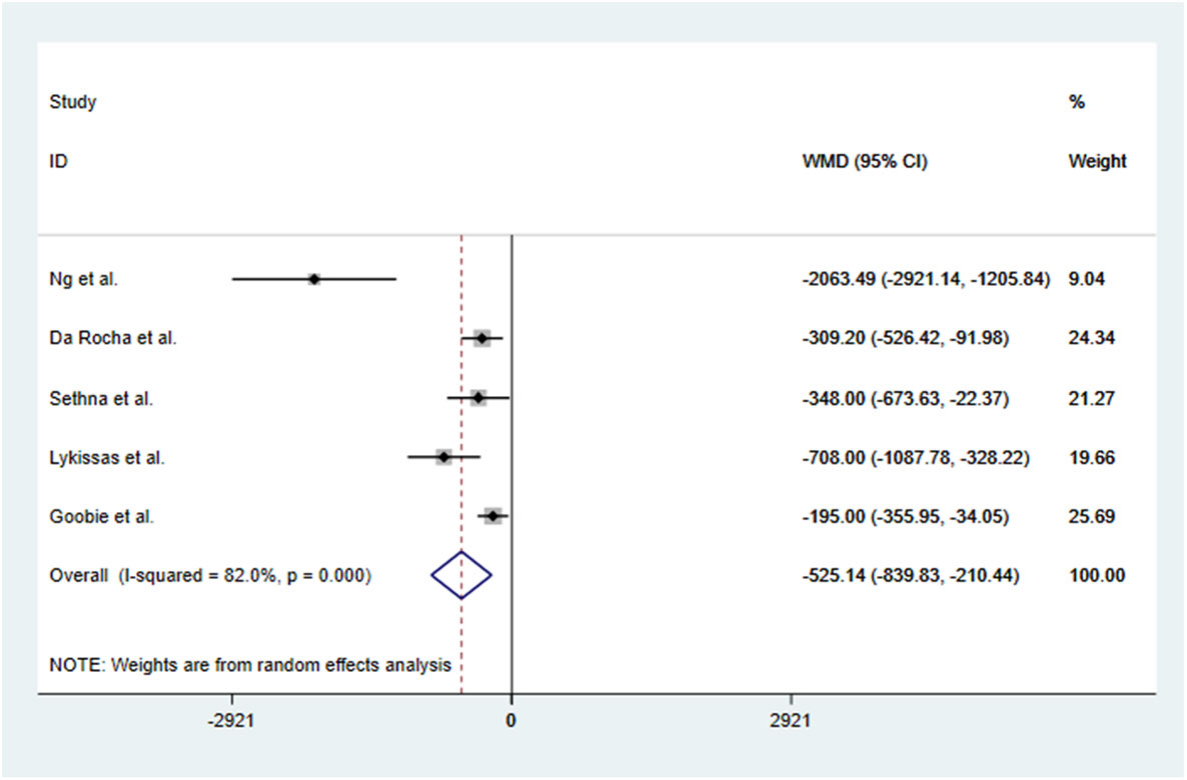
Forest plot of the effect of TXA on estimated blood loss

**Fig. 4:**
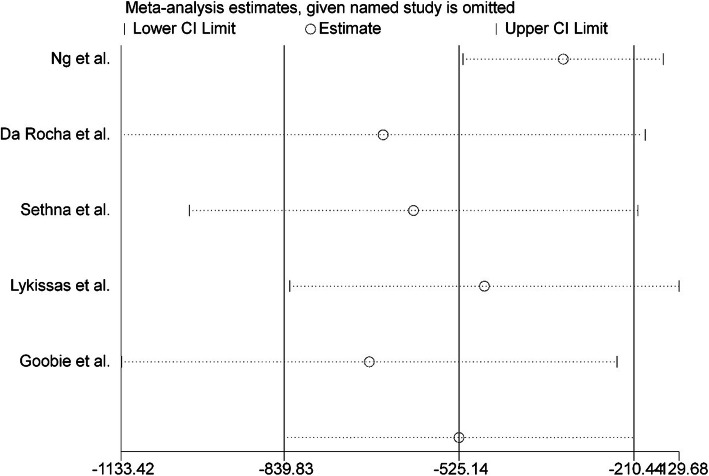
Sensitivity analysis of blood loss

The WMD of blood loss and blood transfusion in the TXA group and placebo group were extracted. Significant heterogeneity was found between the studies with *P* = 0.000 and *I*^2^ = 82%, and a random effects model was used for continuous outcomes. The results showed that a high dose of TXA was effective in reducing blood loss during adolescent idiopathic scoliosis surgery, and the difference was statistically significant [random model: WMD = − 525.14 ml of blood loss, 95% CI ranged from − 839.83 to − 210.44, *P* = 0.000] (Fig. 3). The dosage helped to minimize the risk of bleeding-related hemodynamic instability. To assess the stability of the result of the five studies, a sensitivity analysis was performed by omitting each individual study in turn. Since there was no alteration in the results, they appear to be reliable. The sensitivity analysis is shown in Fig. 4.

### Calculation of surgery time

**Fig. 5:**
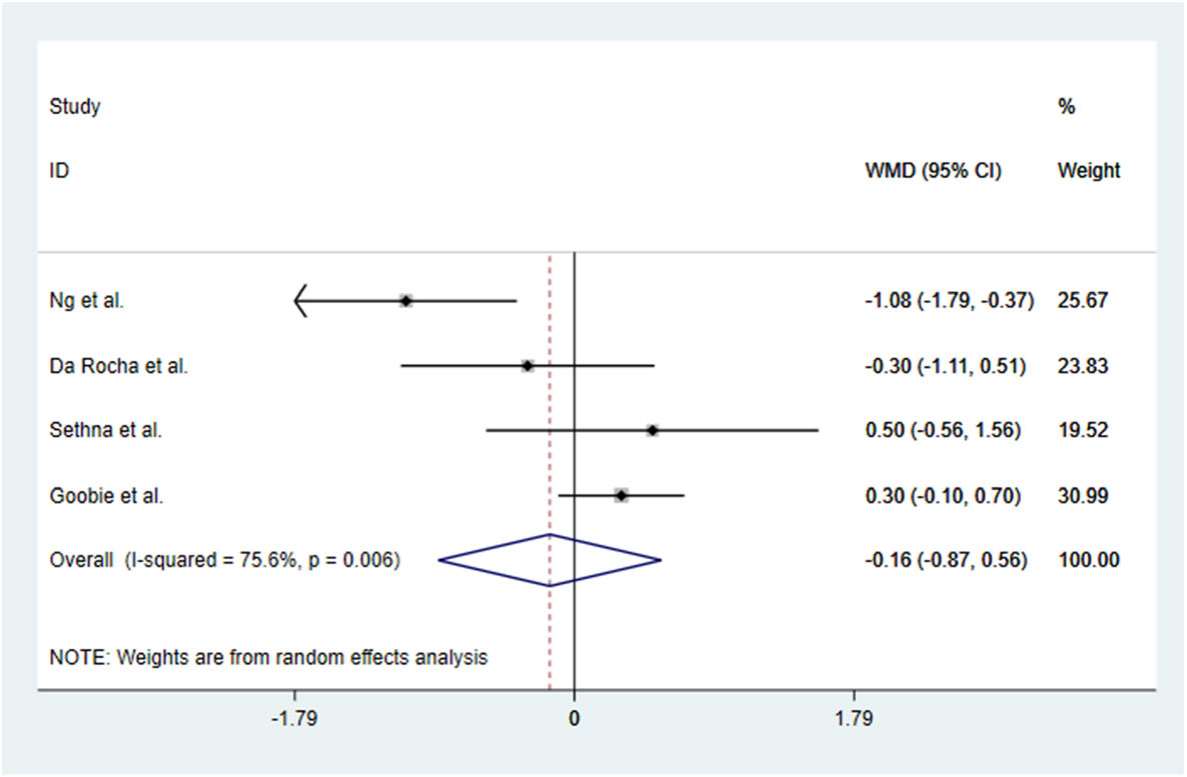
Forest plot of operative time

**Fig. 6:**
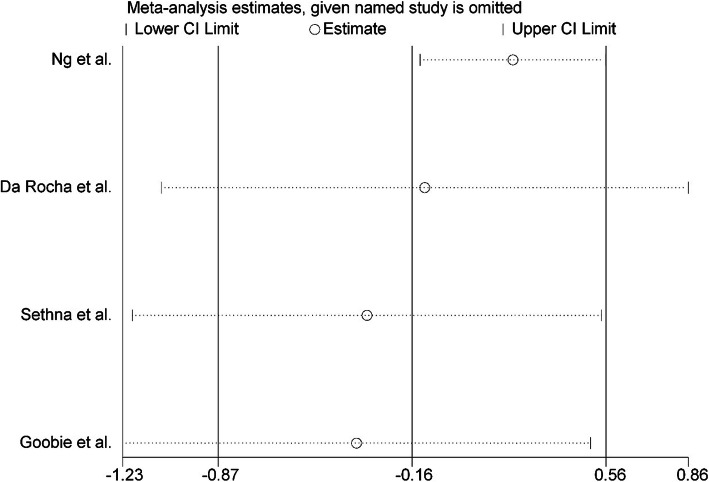
Sensitivity analysis of operative time

Four out of the five studies had reported surgery time. The times reported in the TXA group ranged from 4.0 ± 1.2 to 7.28 ± 2.04 h and in the control group from 4.3 ± 1.4 to 8.36 ± 1.43 h. The TXA seems to have minimized the surgery time regardless of the level of spinal fusion. Blood loss and surgery time are directly proportional to the level of spinal fusion but depend upon the surgeon, the techniques used, and the patient’s position. Significant heterogeneity existed between the five studies with *P* = 0.006 and *I*^2^ = 75.6%, and they all used a random effects model for continuous outcomes. All the statistically analyzed results indicated that the TXA group had reduced surgery times as compared with the placebo or control group [WMD = − 0.16, 95% CI − 0.87, 0.56, *P* = 0.006] (Fig. 5). To assess the stability of the results of the studies, a sensitivity analysis was performed by successively omitting each individual study. There was no alteration in the results, which shows they were reliable with respect to surgery time. The sensitivity analysis is detailed in Fig. 6.

### The effects of TXA on reducing the need for blood transfusion or cell salvage

All five studies reported that TXA reduced the blood loss, which directly minimized the transfusion rate as compared to the control or placebo group. da Rocha et al. demonstrated that the use of TXA reduced blood transfusion by 18.8% compared to the control group [11]. Lykissas et al. reported there being a significant difference between the TXA and the control groups (426 ± 247 mL vs 740 ± 604 mL, *P* = 0.022) [13]. Similarly, Ng et al. reported that patients received a significantly lower volume of cell saver blood transfused back (0.6 L vs 1.7 L, *P* < 0.01) [10]. Finally, Goobie et al. demonstrated that TXA diminishes blood transfusion by two thirds, i.e., median 1 unit vs. 3 units, *P* < 0.001 [14].

### The side effects of TXA during intraoperative and postoperative periods

Despite the high dose of TXA used to reduce blood loss in adolescent idiopathic scoliosis surgery, none of the five studies mentioned any complications, such as a thromboembolic event, during the intraoperative and postoperative period. However, an incidence of 1.3 to 3.8% of clinical convulsive seizures was reported after the administration of a high dose of TXA in cardiopulmonary bypass and open heart-chamber cardiac surgery, especially in older patients [16, 17].

## Discussion

Massive hemorrhage in spinal surgery can lead to unstable vital signs of patients, increase the risk of hypoperfusion of important organs, and increase the probability of hematoma formation, nerve compression, secondary operation, allogeneic blood transfusion, and other events, which can seriously affect the surgical effect and prognosis of patients. Therefore, the control of perioperative bleeding in spinal surgery has always been an important concern of spinal surgery teams and anesthesiologists. There are various natural antagonists of plasminogen in the blood circulation, such as antiplasmin. Under normal circumstances, the activity of anti-fibrinolytic substances in the blood circulation is many times higher than that of fibrinolytic substances, so fibrinolytic bleeding will not occur. However, these antagonists cannot block the plasmin that is activated by activators absorbed by the fibrin network. Plasmin can cleave the arginine and lysine peptide chains of fibrinogen in a neutral environment, leading to the degradation of fibrin, and the dissolution and bleeding of blood clots. TXA is a synthetic derivative and homolog of lysine, which can pass through the blood cerebrospinal fluid barrier. It has a high affinity with the lysine binding site of fibrinogen, which can block the interaction between fibrinogen containing lysine residues and heavy chains of plasmin, preventing fibrinolytic enzymes from degrading fibrin, and, thereby, playing a hemostatic effect in the perioperative period of spinal surgery [2]. At present, spinal surgeons have also begun to use tranexamic acid to reduce perioperative blood loss and have achieved good results. Although some meta-analyses have been done on low-dose TXA for posterior spinal fusion, there is no study of the efficacy and safety of high-dose TXA in adolescent idiopathic scoliosis surgery.

In this meta-analysis, a total of three RCSs and two RCTs were included, involving 334 patients. The outcomes of this study showed that a high dose of TXA reduced blood loss during adolescent idiopathic scoliosis surgery (WMD = − 525.14, 95% CI − 839.83, − 210.44, *P* = 0.000), the TXA group had reduced surgery times when compared with the placebo or control group (WMD = − 0.16, 95% CI − 0.87, 0.56, *P* = 0.006), and the difference was statistically significant.

The most significant finding of this study of high-dose intravenous TXA is that it can lead to a reduction in blood loss and the transfusion rate. During scoliosis surgery, there is a risk of significant perioperative bleeding causing the formation of spinal hematoma, which may result in spinal cord or cauda equina compression [18, 19]. However, none of the studies in this meta-analysis, nor other studies that are not included, mention any complications, such as thromboembolic events or seizures, during the operation or the postoperative period [10–14]. Nevertheless, despite the positive outcomes for efficacy and safety, Myles et al. mention that TXA has an associated risk of postoperative seizures [6]. The reduction in blood loss depends on the maintenance of mean arterial pressure within a band and depends on the surgeon’s technical skills for pedicular instrumentation and hemostasis [11]. Most of the studies to date were focused on hip replacement surgery, knee surgery, and cardiac surgeries, and some studies have focused on the field of gynecology and obstetrics. All of the studies [10–14] mentioned that the intraoperative blood loss was reduced by 41–51% for those that used TXA as compared to a placebo or non-TXA group. Popovsky et al. [20] mentioned that autologous donors were twelve times more likely than general donors to experience an adverse event that required hospitalization. In the literature, it was reported that an autologous blood transfusion also has complications that depend on the quantity of blood used [13, 20]. Even preoperatively donated autologous blood has an inherent level of risk [13]. Lykissas et al. [13] stated that aprotinin was associated with cardiac or renal failure, myocardial infarction, stroke, or death. Xie et al. [21] mentioned that with a high dose the previously studied low dose showed no signs of renal toxicity, seizures, DVT, or MI. Based on our five studies and the above results, we can conclude that a high dose of TXA is safe and effective, but an optimum dose should be recommended to avoid over-generalization.

There are a number of other studies concerning TXA, some of which support our findings while others do not. Johnson et al. [2] stated that a high or very high dose transfusion is an especially high risk for hospital-acquired infections, thrombotic events occurred 4–5 times more commonly than a renal, respiratory, or ischemic event, and mortality increased linearly over the entire dose range and exceeded 50% after 50 erythrocyte units. Chiem et al. [22] reported that tranexamic acid caused an anaphylactic reaction in a 15-year-old patient scheduled for posterior spinal fusion, and another case of an anaphylactic reaction involved a 72-year-old male, who underwent coronary artery bypass graft surgery after a bolus of tranexamic acid. On the other hand, Hui et al. [23] reported that a high dose of TXA can reduce intraoperative allogeneic blood transfusion, operative time, and cell salvage transfusion compared to a low dose. Perel et al. [24] mentioned that TXA can reduce mortality in emergency and urgent surgery, which is associated with a high risk of bleeding and death. In a randomized double-blinded placebo-control trial, Goobie et al. [14] demonstrated that a 50 mg/kg bolus and a 10 mg/kg/h maintenance dose were effective in reducing blood loss in PSF surgery, and none of the TXA group patients required allogeneic blood transfusion or suffered any side effects. However, Bosch et al. [25] in a comparison of coagulation profiles reported that TXA use in PSF in AIS patients decreased the transfusion rate but showed no significant change in blood loss. Zhong et al. [26] demonstrated that TXA was effective in reducing surgical time, intraoperative blood loss, and allogeneic blood transfusion without any increase in complications in AIS. Research, on the whole, seems to point to the benefits of using TXA, since the reduction of blood loss during the intraoperative and postoperative periods has a direct effect on minimizing hospital stays and any financial burden, as well as increasing the likelihood of a good prognosis. Thus, it would appear that in scoliosis surgery, the use of TXA is safer and cheaper than other antifibrinolytics.

## Limitations

This meta-analysis has a number of limitations. First, although it was the first meta-analysis to date to study high-dose TXA administration, there were only three RCSs and two RCTs. Second, the subgroup analysis could not be done due to the limited sample size, and this indirectly influenced the final result. Thus, further research is necessary. Third, the follow-up of this meta-analysis was short-term, and another longer-term study on the safety of tranexamic acid is still required. Lastly, there needs to be more research into different doses of TXA and different time duration of bolus doses.

## Conclusions

A high dose of intravenous tranexamic acid reduced blood loss during adolescent idiopathic scoliosis surgery and was connected with no significant thromboembolic events. Therefore, a high dose of TXA seems to be effective and safe for adolescent idiopathic scoliosis surgery. However, further studies with a larger sample size are required to confirm the safety of a high dose of TXA for such surgery.

## Data Availability

We declared that materials described in the manuscript, including all relevant raw data, will be freely available to any scientist wishing to use them for non-commercial purposes, without breaching participant confidentiality.

## Abbreviations

CI: Confidence interval
PSF: Posterior spinal fusion
TXA: Tranexamic acid
RCT: Randomized control trial
RCSs: Retrospective cohort studies
NOS: Newcastle-Ottawa Scale
RR: Relative risk
CIs: Confidence intervals
